# Retraction note: Rituximab-conjugated, doxorubicin-loaded microbubbles as a theranostic modality in B-cell lymphoma

**DOI:** 10.18632/oncotarget.14897

**Published:** 2017-01-30

**Authors:** Shoubing Zhou, Xiu Zhang, Cailian Wang

**Retraction**: Due to the authors’ negligence during the preparation of this manuscript for submission, it contains a plethora of data errors. Authors sincerely apologize for their mistakes and wish to retract their manuscript.

**Footnotes**: The online version of the original article can be found under doi.

Original article: Oncotarget. 2017; 8(3):4760-4772. doi: 10.18632/oncotarget.13587.

